# Rational Steps in the Scientific Treatment of Nervous Prostration, Headache and Neuralgia

**Published:** 1900-04

**Authors:** Ambrose L. Ranney

**Affiliations:** New York City


### RATIONAL STEPS IN THE SCIENTIFIC TREATMENT OF NERVOUS PROSTRATION, HEADACHE AND NEURALGIA.

By AMBROSE L. RANNEY, M. D., New York City.

"NONE are so blind as those who won't see!" There are many in the medical profession, to-day as in the past, who stick to antiquated methods; who unfortunately regard all innovations as either nonsensical or useless; who scoff at all facts which conflict with their pet theories and methods; who refuse to even try what they have groundless prejudices against; who talk learnedly in societies and elsewhere about the results of investigations by themselves or others that can never have been made intelligently, if at all; and who write as fluently as they talk derogatory to an important field in medicine that they have little if any technical knowledge of, no sympathy with, no experience in, and whose results (in such hands) must of necessity have been unsatisfactory.

Everyone who has worked intelligently and faithfully for years in the scientific study of the eye-muscles knows today that the opinion of any one on their anomalies must be of little value—no matter how great the reputation of the man—if he has neither the "phorometer" nor "tropometer" as a part of his office equipment, nor any records of the power of each individual muscle and the arcs-of-rotation of each eye upward, downward, inward and outward.

There is indisputable proof today that "crossed" eye toward the nose or temple—chiefly the former—may in some cases be made parallel with its fellow by simply lowering or raising one or both eyes. Such cases were not understood five years ago. They tend today to explain why many operations for "squint" have, in the past, caused bad over corrections or yielded negative results. It is known today that the muscle at fault in "cross-eye" is not always the muscle that to the casual observer appears to restrict the natural movements of the eye. The cutting of the wrong muscle may sometimes give the operator who follows antiquated methods of research before operation—and the patient as well—subsequent food for reflection and a vast amount of regret.

The sad results, viewed from the standpoint of suffering humanity, that are entailed by indifference and prejudice in men of scientific reputation cannot be estimated.

By giving expression to others of their opinion concerning what they have not properly and patiently investigated themselves or will not see, thousands of sufferers are doomed to a life of misery.

Such patients naturally believe that abnormal eye factors in their own case have been sought for by the latest methods and found to be absent by one who stands high in his profession. They quote to their friends the positive assertions of him—whom they believe infallible—that eye muscles are not worth investigating;" that "all deviating tendencies of the eyes are invariably due to errors of refraction;" that "Javal's instrument has been used, and that settles the question forever;" and other similar expressions, indicative either of inexperience, bigotry, or prejudice, that too often come to my own ears.

Perhaps the most common experience that I personally encounter in my office, is to have patients tell me that either their doctor or some oculist whom they have consulted has said to them that "their eye trouble is the result of their physical weakness and not a cause; that the relief of the eye trouble can have nothing to do with their recovery and that all statements to the contrary are not supported by facts."

It is for the purpose of demonstrating the counter-proposition; of showing that "eye-strain" may indirectly be the cause of obscure disease and not its result; and of turning, if possible, the channel of medical thought so as to benefit suffering humanity, that I deem it wise to raise my voice again in defence of a method of treatment that will often accomplish what drugs will not, and that is based upon science rather than therapeutical speculation and empiricism.

From my late work, entitled, Eye-Strain in Health and Disease, F. A. Davis Co., publishers, Philadelphia, 1897, I quote the following paragraphs relative to the effect of a leakage of nerve-force upon the predisposition of certain persons to obscure diseases:

"* We are all exposed—from birth until death— to the presence of certain atmospheric germs or microbes. Many forms of these are constantly being introduced into our bodies through the air, our food, and the water we drink. Constantly, through life, are certain toxic materials also being formed within the human body and eliminated through the kidney, liver, intestinal tract, etc.

In some subjects, when the most favorable conditions for their retention, absorption, and multiplication exist, do these microbes and toxic substances hasten the dissolution of life; while in others that possess a reactive power capable of combating the elements of disease, life goes on in spite of microbes with its normal growth and decay.

We are forced to admit that seeds sown by the farmer as well as those of the weed-carried and distributed by the wind flourish best in soil that is properly prepared for their reception and development. Is it therefore irrational to assert that any of the underlying causes of nervous debility, any constant sources of excessive and unnatural nervous expenditure, any hereditary condition that creates an uninterrupted leakage of nerve-force, may be an important factor in fitting the human soil for the seeds of disease that are ever present and ready to take root?

Should investigation end whenever microbes or toxic materials are discovered in the human economy? Should the causes of a diseased condition be regarded as determined so long as the underlying factors that enable this cause to exist and flourish are ignored? Which is the most important in medicine—to ascertain what adventitious agent can do damage under favorable conditions, or to so improve the nervous power of a patient as to enable the body to resist the seeds of disease?

* So long as we are incapable of fighting our foe (when once established with a firm foothold within the human economy), what have we gained from the standpoint of the sufferer by simply knowing the name, exact appearance, or seat of the enemy?

I would not be construed as decrying the scientific results obtained by careful, painstaking bacteriological or pathological research. They undoubtedly tend to furnish the medical practitioner with some knowledge, after the chaff has been eliminated and the wheat carefully collected, that may in time aid in the prevention of disease, and possibly facilitate its cure. But, while these investigations are going on, while with great rapidity theories are being hatched and exploded, while the views of today may not be those of tomorrow, is it not the duty of every earnest practitioner of medicine to listen with attention to clinical facts that are capable of absolute verification, that are often frought with immediate results to suffering humanity, and that open a wide field for the treatment and relief of obscure nervous diseases?

The ten cases which I desire to present to the reader in this connection are of special interest to the general practitioner of medicine because many forms of drugs had been employed by physicians of repute upon these sufferers (prior to my treatment of their eyes) without any appreciable benefit of a permanent character. I am therefore justified, I think, in claiming that simply by the arrest of a leakage of nerve-force (which had been entailed upon these patients by errors of focus and certain anomalies of adjustment of the eyes) I gave nature for the first time a chance to reassert herself in these patients, and I thus restored indirectly the condition of perfect health.

To anyone who delves in the medical literature of the past, and who reads the theories that have flourished and been forgotten, it becomes at once apparent that the tendency of this age is to seek for the causes of disease, and, as far as possible, to remove them.

The argument that is advanced here is no longer a theory. It is supported by well-established physical laws and sustained by physiology. Furthermore, the results obtained when the abnormal eye factors have been scientifically determined and removed, can be shown to be almost instantaneous, and somewhat startling, in many of the cases thus treated.

In the reported cases which follow, some technical terms are employed that may require explanation to the general practitioner, although they would be easily understood by the oculist. These are comprised in the following table:

| | |
|---|---|
| Terms related to the focus of the eye (*refractive terms.*) | HYPERMETROPIA *(far-sightedness)*. A *shallow eye* (from the front to the back), causing an imperfect focus of objects. |
| | MYOPIA *(near-sightedness)*. An *elongated eye* (from the front to the back), causing an imperfect focus of objects. |
| | ASTIGMATISM. An *irregularly curved cornea* or *lens*, causing distortion of images on retina. |
| | EMMETROPIA. A *perfectly constructed eye.* |

| | |
|---|---|
| Terms related to the muscles which move the eyes (*muscular terms.*) | ESOPHORIA. A tendency of one or both eyes to deviate toward the nose. |
| | EXOPHORIA. A tendency of one or both eyes to deviate toward the temple. |
| | HYPERPHORIA. A tendency of one eye to rise above the level of its fellow. |
| | ANAPHORIA. A tendency of both eyes to assume too high a plane. |
| | KATAPHORIA. A tendency of both eyes to assume too low a plane. |
| | HETEROPHORIA. Any abnormal adjustment of the eye muscles. |
| | ORTHOPHORIA. Normal adjustment of the eye muscles. |
| | ADDUCTION. The power of the internal muscles of the eyeballs. *It varies in health between 25° and 60°.* |
| | ABDUCTION. The power of the external muscles of the eyeballs. *It should be 8° in health.* |
| | SURSUMDUCTION. The power of the vertical muscles of the eyeballs. *The right and left should be alike.* |
| Various forms of glasses employed by oculists. | SPHERICAL. Ground upon a *convex or concave sphere.* Used to correct hypermetropia and myopia. |
| | CYLINDRICAL. Ground upon a *convex or concave cylinder.* Used to correct astigmatism. |
| | PRISMATIC. *Two plain surfaces of glass meeting at an angle.* The thick side is termed the base of the prism. Used to relieve errors of adjustment of the eye muscles. |

One very important fact is brought out in the clinical history of some of the cases here reported, to which the attention of the reader should be forcibly drawn, viz.: that the correction of the refraction, of some of these patients by glasses had been very thoroughly done by competent oculists before I saw the patients, without any appreciable relief to their nervous symptoms.

This point is made here because it has been, and still is asserted by a few oculists of repute, that "all muscular defects in the orbits are dependent upon and always caused by errors of refraction." Such a statement is too absurd and untenable to be discussed seriously. It can be proven so beyond the possibility of a doubt, by any one who has had a large experience in treating anomalies of the ocular muscles.

Cases I. II. and III. alone ought to show to any unprejudiced mind that the refraction of the patient was independent of the muscular defects. Yet these defects entailed eye-strain to a degree that was destroying the general health—and still they had been overlooked or ignored.

Case I.—*Nervous prostration, with melancholia and complete physical collapse.*—Mrs. C., married; age, 35. This patient had always been near-sighted and astigmatic. She had worn glasses since she was a girl. Like most suffers, who are victims to the peculiar form of anomaly of the eye-muscles that she disclosed, she had been troubled more or less from childhood with headache. She had always been rather a delicate woman; but in latter years she had gradually become unable to endure shopping, attendance at the theatre, or traveling without marked physical exhaustion and suffering. Although the wife of a very prominent lawyer and blest with all the surroundings of wealth, she also commenced some five years before I saw her to suffer greatly when using her eyes for any length of time. For this difficulty she was treated by various oculists, but without any marked benefit. Gradually her physical condition began to run down; in spite of tonics, massage and electricity. With this loss of strength an alarming change in her mental condition developed. From being a buoyant, happy wife, she became almost a melancholiac. From this condition it was impossible at times to arouse her. She was obliged in consequence to give up almost all social duties and amusements; and the accompanying physical weakness kept her confined to the house for a greater portion of the time.

Her family and physician had become justly alarmed before I saw her lest her mental condition might assume an incurable type. She was sent to me to ascertain chiefly if I could find any cause for her nervous collapse and mental depression. A careful examination of her eyes showed that she was wearing proper glasses for her near-sightedness and astigmatism; but that a high degree of "latent" esophoria existed. During the few days that she wore prisms (for the investigation and partial correction of this trouble) she experienced such immediate and marked relief both in respect to her eyes and physical weakness that she and her family both welcomed with delight a possibility of a radical correction of the esophoria and the cessation of its attendant eye-strain. During the six weeks she was in New York under my care, a button-hole tenotomy was performed by me on both internal recti, with the most gratifying results from both the physical and eye-standpoint. The improvement in her mental condition was immediate. Before she left this city to return home, she was able to walk long distances, do moderate shopping without fatigue or headache, attend places of amusement and use her eyes for reading with more comfort than for many years. It is now over a year since she returned home. During that time she has entirely regained her former cheerfulness and health. She is today regarded by her physician and family as entirely cured. Her headaches have entirely ceased. She is no longer car-sick. She is able to use her eyes moderately without pain or inconvenience. She has gained greatly in weight and strength, and is again prominent in all social affairs.

Eye-tests were lately made in my office to see if her eye-muscles required further treatment. It was found that perfect "orthophoria" existed. The balance established by two painless steps was perfect; and no trace of the old tendency of the eyes to cross could be discovered.

Case II.—*Severe headaches of a chronic type with frightful exacerbations, that had yielded to no previous treatment.*—Dr. E., age 44; married. This patient is one of the leading surgeons of America. For many years he had been the victim to frequent and frightful headaches, which absolutely prevented all surgical work for several days at a time. On his first visit to my office he reported that he "had not been free from headache sixty days in the past year." For years he had tested thoroughly every form of therapeutic measure for relief of pain. The results had been absolutely negative. Like many others he had been somewhat prejudiced against the views which I hold; and he had been strengthened in his prejudice by the statements of several oculists of prominence that his eyes had nothing to do with his headaches.

When he first came under my care, the most careful examination of his eyes under atropine failed to show much if any refractive error; and to this day he can not tolerate any glass with comfort, except a weak spherical glass for his surgical work, as the result of age. He disclosed, however, after some days of repeated tests and observation, a high degree of "latent" esophoria, and also some tendency of the left eye to assume a higher plane than the right eye—left hyperphoria.

Within the space of one year, I performed a button-hole tenotomy upon both of his interni and I also lowered his left eye. Since that time he has passed about eighteen months with practically no pain; has never lost for one day his ability to do surgical work; and he has quite lately assured me that he regarded himself as perfectly cured. He is able now to use his eyes with absolute comfort at all times; and without the headaches that were sure to follow eye-fatigue, before his eye-muscles were properly balanced.

From a scoffer he has now become a thorough convert to the importance of seeking for eye-strain whenever a tendency to headache exists.

Case III.—*Excruciating Facial Neuralgia, incurable by drugs and leading to suicidal impulses.*—Mr. C.; married; aged 35; hotel manager. This patient came to place himself under my care after years of the most acute suffering that it is possible to describe. He had suffered for ten years with almost constant facial neuralgia, involving chiefly the left side. So long as the pain was moderate, he could endure it. Acute paroxysms would come on, however, very frequently and at unexpected times without apparent cause. The intensity of the pain at such times in the teeth, cheek and orbit would almost deprive him of reason until morphine could be administered hypodermatically.

He was treated for some years for these attacks by a large number of the leading specialists of New York City without any relief.

oculist of New York, who prescribed glasses to correct about one and a half dioptres of hypermetropia and a slight degree of astigmatism. These he had worn constantly for about three years but no appreciable benefit had been experienced from wearing them, except that he "thought he saw a little better with them." He had been for many months under the care of the late Prof. E. C. Seguin, the distinguished neurologist, who experimented upon him with almost every narcotic known. At one time, aconitia was pushed, under Dr. Seguin's personal supervision, until aconite poisoning appeared. The patient could not at one time talk plainly from the effects of this drug and the tongue and throat became almost paralyzed for several days. The intensity of the neuralgic paroxysms at times made his family fear suicide, and the patient learned to administer morphia and atropia by the syringe to himself when he felt that the attacks were getting beyond human endurance.

Throughout six or seven years of such suffering, this victim to neuralgia consulted almost every specialist of note in his vain efforts to obtain relief—but in despair over his failure, he became my patient at the solicitation of a friend who had been personally cured by eye-treatment.

On examination of this patient's eyes, I found that Dr. Loring had given him glasses that were a full correction for all his refractive errors; but like many other oculists, he had not investigated the eye-muscles, which were seriously at fault. He disclosed a very moderate degree of esophoria at the first examination, but later on I found him to be a victim to "latent" esophoria of a high degree. Within two months, I performed a graduated tenotomy upon both interni of this patient. His neuralgia ceased entirely within a week from the date of the first operation, and he has been entirely free of neuralgia for the past ten years.

About two years ago this patient called upon me while in New York and stated that "his marvelous restoration to perfect health by so simple and painless an operation had been considered a miracle by his friends and family." His eye-tests showed no marked change in his refraction, except that he needed a stronger glass for close work, which was ordered. I found, however, to my delight that the esophoria which originally existed and had caused all his suffering was completely relieved; and he left with the happy assurance that he had no reason as far as I could see to anticipate a return of his old trouble from eye-strain.

Case IV.—*Complete Nervous Prostration with a total loss of emotional and mental control, sleeplessness and headache.*—Miss B., aged 40, single; lecturer.

*Family History.*—One sister was over a year a victim to "complete nervous prostration." Father is a very nervous man.

*Eye Defects.*—Vision $\frac{20}{20}$, without atropine. Under atropine, a latent hypermetropia of +0.75 s. in each eye. Patient had never used a glass for reading, but accepted +1.50 spherical glass. Esophoria

3° (which ultimately, under influence of prismatic glasses, exceeded 7°). Adduction, 23°. Abduction, 5°. R. sursumduction, 1°+. Left sursumduction, 2°. The adducting power later on exceeded 43°, and the abducting power fell below 3°. At no time did homonymous diplopia disclose itself (with or without a red glass).

*History of Case.*—This lady had for some years been doing an excessive amount of mental work. Her profession required an enormous amount of reading. This had been done largely at night. Although small in stature, she had always been vigorous and had taken an unusual amount of exercise. She *had always considered her eyes very strong*, and was loath to believe, when she first came under my care, that her eyes could constitute a factor in her serious nervous condition. Furthermore, she was strengthened in this belief by the fact that she had not long before consulted an oculist of prominence, who had stated that he found no defect requiring treatment or glasses, and who had sent her to one of his friends (a specialist in nervous diseases) for treatment.

The "break-down in her health" began about twelve months before she came under my care. It was attended with an extreme and persistent loss of sleep, loss of emotional control, an utter inability to read or sew (any attempt at the latter always aggravated all her symptoms), a more or less constant headache, an inability to concentrate her intellectual faculties for any length of time, and an aggravated type of mental depression. She feared, and had every apparent reason to fear, that her professional labors were imperiled and that her mind might possibly give way. The neurologist, who endeavored to build her up by tonics, rigid diet, rest and the like, assured her (after some improvement had occurred) that he feared at first that "melancholia" might be the end of the case. At his advice, she spent the summer at the sea shore; but, beyond a certain point, she failed to progress satisfactorily, and her headache and sleeplessness would at times be as bad as ever. Any attempt to prepare herself for her fall engagements would cause a return of her old symptoms to a very marked degree, accompanied by physical weakness, mental fatigue and depression, extreme despondency, and a lack of control over her emotions. After any attempts at study she would frequently lie awake most of the night. This was her condition when she first came under my care.

*Treatment and Results.*—In this case a full correction of the hypermetropia was made for distance, and + 2·00 spherical glasses were given for reading, as she showed some failure of accommodation. Prisms of various strengths were employed over her distance and reading-glasses for about two weeks, and 7° of latent esophoria were found to exist. This was rectified by a graduated tenotomy of one internus and the prisms were then discontinued. During this interval the patient had improved very rapidly, had become very dependent upon the spherical glasses, and become cheerful and hopeful of recovery. She had moreover, entirely regained the normal power of sleep. During this interval she had frequently slept twelve hours without awakening and without recourse to any drug. As atropine had been used during the early part of the treatment, she had been allowed during the two weeks of treatment to use her eyes very little in reading or study. During the following two weeks two degrees more of latent esophoria disclosed itself. For the relief of this defect a prism was combined with the spherical glass worn over the eye which had not been subjected to a tenotomy.

This patient soon became able to fill all her engagements without any return of her bad symptoms. She has since read and studied at night, attended church and places of amusement that previously she dared not attend, has accepted more work than for some years past, and has continued to sleep well and enjoy perfect health. She has taken no medicine, nor has she been restricted by me in her diet or in any other way. Her reading-glasses have been increased to +2.50 s.

The records of her case show that a graduated tenotomy of the internus of the uncorrected eyes will eventually have to be performed, in order to properly adjust the balance between the two eyes.

During one of her last visits this patient said: "I think I am stronger today and have better health than I have had for many years. I certainly do my work with less fatigue, and enjoy things that my ill-health has previously debarred me from."

*(To be concluded in the May number.)*

